# Identification of Orthotropic Elastic Properties of Wood by a Synthetic Image Approach Based on Digital Image Correlation

**DOI:** 10.3390/ma15020625

**Published:** 2022-01-14

**Authors:** João Henriques, José Xavier, António Andrade-Campos

**Affiliations:** 1 TEMA, Department of Mechanical Engineering, University of Aveiro, Campus Universitário de Santiago, 3810-193 Aveiro, Portugal; joaodiogofh@ua.pt (J.H.); gilac@ua.pt (A.A.-C.); 2 UNIDEMI, Department of Mechanical and Industrial Engineering, NOVA School of Science and Technology, NOVA University Lisbon, 2829-516 Caparica, Portugal

**Keywords:** wood, inverse identification, full-field measurements, DIC, synthetic images, FEMU, orthotropic elasticity, compression tests

## Abstract

This work aims to determine the orthotropic linear elastic constitutive parameters of *Pinus pinaster* Ait. wood from a single uniaxial compressive experimental test, under quasi-static loading conditions, based on two different specimen configurations: (a) on-axis rectangular specimens oriented on the radial-tangential plane, (b) off-axis specimens with a grain angle of about 60° (radial-tangential plane). Using digital image correlation (DIC), full-field displacement and strain maps are obtained and used to identify the four orthotropic elastic parameters using the finite element model updating (FEMU) technique. Based on the FE data, a synthetic image reconstruction approach is proposed by coupling the inverse identification method with synthetically deformed images, which are then processed by DIC and compared with the experimental results. The proposed methodology is first validated by employing a DIC-levelled FEA reference in the identification procedure. The impact of the DIC setting parameters on the identification results is systematically investigated. This influence appears to be stronger when the parameter is less sensitive to the experimental setup used. When using on-axis specimen configuration, three orthotropic parameters of *Pinus pinaster* ($E_{R}$, $E_{T}$ and $\nu_{\mathrm{RT}}$) are correctly identified, while the shear modulus ($G_{\mathrm{RT}}$) is robustly identified when using off-axis specimen configuration.

## 1. Introduction

Engineering materials based on renewable and recyclable natural resources are regaining momentum towards policies and practices of sustainable green economy. Wood and wood-based products are an important class of these materials, with a long-term increase in demand worldwide [1]. Wood is a complex, heterogeneous, and hierarchical biological material. Therefore, it is quite complex to mathematically describe or characterise from both numerical and experimental points of view. At the macroscropic scale, where the concept of clear wood is introduced, a mechanical model based on an anisotropic behaviour is typically accepted, assuming three orthotropic material directions: the longitudinal direction (L) along the tracheids, the radial direction (R) parallel to the rays, and the tangential direction (T) to the annual growth rings [2]. However, several degrees of heterogeneities exist. On the one hand, locally, in earlywood (EW) and latewood (LW) cellular tissues, different physical and mechanical properties can be identified at the growth ring scale [3,4]. On the other hand, globally, radial and longitudinal spatial gradients can be observed at the structural (stem) scale [5,6].

The use of wood and wood-based products for structural applications implies the understanding of the material behaviour under mechanical loadings. Although the use of finite element analysis (FEA) to simulate processes is well established, the calibration of material constitutive models is still a bottleneck in terms of feasibility. The characterisation of material parameters is critical in computer-aided engineering systems to properly replicate the material behaviour [7,8,9]. Experimental mechanics typically rely on surface deformation data. Classical mechanical tests are based on a uniform or linear stress field at the gauge section, under simplified assumption, to allow the derivation of closed-form solutions for the inverse problem of determining mechanical properties from measurements (geometry, load and strain) [2,10,11]. Nevertheless, only a limited number of parameters can be identified per test configuration, due to the simplified geometry and loading scenarios. Therefore, for fibre-based or advanced composite material, a large number of independent test methods are typical required to obtain all the constitutive parameters. This approach proves to be time-consuming and costly [12]. Moreover, homogeneous strain fields are required in each test, to measure in-point displacements and strains using extensometers and strain gauges [13,14]. These methods also do not address the problem of spatial variability of mechanical properties caused by the natural heterogeneity of biological materials, such as wood [2,15].

Recent advances in digital imaging technology have enabled the development of novel optical techniques in solid and fluid experimental mechanics [16,17,18]. These techniques are being increasingly used in diverse applications since these are contact-free and provide full-field measurements. Among these techniques, digital image correlation (DIC) [19,20,21,22,23,24,25,26] and the grid method [27,28,29] have been exponentially used in the recent past due to their simplicity and good balance between spatial resolution and accuracy. The 2D-DIC subset-based correlation technique is highlighted and is used in this work. In this technique, the DIC setting parameters, such as subset size, step size, strain interpolant, strain interpolation and window size can significantly impact the measured strain fields and the absolute error of measurements [2,30,31], and therefore directly influence the identification of material constitutive parameters [32]. The selection of these settings should not be overlooked when performing displacement and strain measurements through DIC, being particularly important in heterogeneous materials, such as wood, where deformation gradients are expected due to the annual growth ring structure.

The development of full-field measurement techniques has allowed a new insight in the experimental characterisation. These techniques have the potential to reduce the number of experimental tests required to fully characterise a material, given that a heterogeneous test configuration is used, producing heterogeneous stress and strain fields in the specimen so that all material properties take a role in the mechanical behaviour. Furthermore, it is appropriate for addressing current open issues on the identification of mechanical properties for heterogeneous materials, such as the spatial variability of mechanical properties over the region of interest (ROI), in materials such as wood or composites [2]. When compared to classical experimental tests, full-field measurement techniques bring far more versatility and can provide incredibly detailed experimental data when using complex test configurations, thus allowing the identification of more constitutive parameters with a single test, and therefore reducing the number of experimental tests needed. To take advantage of full-field measurements, several inverse identification approaches have been proposed [33], i.e., the equilibrium gap method [34], the constitutive equation gap method [35], the finite element model updating method (FEMU) [36,37,38] and the virtual fields method (VFM) [39,40,41,42]. The VFM is a well-established approach for characterising material properties directly from full-field measurements using a specific application of the principle of virtual work [13]. The FEMU involves the development of a finite element (FE) model of the experimental test and the minimisation of a cost function based on the difference between measured and calculated values through an iterative optimisation process.

The experimental validation of FEA is critical to develop the credibility in numerical model predictions for engineering design. Traditionally, the data from experimental DIC measurements and FEA would be directly compared. However, the experimental characterisation is evolving to a new paradigm [43]. The direct comparison between the numerical and experimental DIC data can lead to differences being falsely associated with model inaccuracies. This can be due to different aspects, such as the different filtering of regions of high strain gradients, differences in data locations and strain formulations, or different spatial cut-off frequencies (the numerical mesh can be further refined according to user details, while the DIC data points are fixed by the camera spatial resolution) [44]. Recently, the photomechanical experimental community is focusing on an alternative approach to compare the differences between the full-field maps from the experimental DIC and the numerical results. This alternative approach involves using the numerical results, such as the FEA displacements and the mesh data, to synthetically deform an experimental image of the specimen with the speckle pattern [45]. The FEA-based synthetic image can then be processed using DIC with the same setting parameters used for the experimental measurements, guaranteeing that both FEA and experimental results have the same spatial resolution, filtering and strain formulation, therefore removing the differences in the full-field maps comparisons that are not due to inaccuracies in the material constitutive model calibration. Furthermore, this approach can also distinguish some pattern-related image artefacts issues from actual constitutive model issues. This is primarily due to the inclusion of these image artefacts issues in the DIC-levelled FEA data, since it involves images of a real DIC speckle pattern [44].

The goal of this work is to identify the linear elastic orthotropic constitutive parameters of *Pinus pinaster* Ait. using uniaxial compression tests under quasi-static loading conditions, with on-axis rectangular specimens oriented on the radial-tangential (RT) plane and off-axis specimens at an orientation to the grain of 60°(RT plane). The influence of the test configuration on the identifiability of certain material parameters is also investigated. Several images of the experimental tests were captured using a digital camera and then processed using DIC with different settings to evaluate the influence of DIC settings on the identified parameters. Heterogeneous full-field displacement and strain maps with strain gradient at the wood growth ring structure were measured and used to determine the material properties, such as modulus of elasticity, *Poisson*’s ratio, and shear modulus. A FE model was developed considering wood as an orthotropic homogenous material and the numerical results were used to synthetically deform the reference experimental image, further processing this synthetic image with the selected experimental DIC settings, resulting in DIC-levelled FEA results. The FEMU method was used for the identification process, which involves the minimisation of a cost function that represents the difference between the experimental and numerical results, including the load and strain fields, resulting in four calibrated material properties determined.

## 2. Materials and Methods

**Figure 1:**
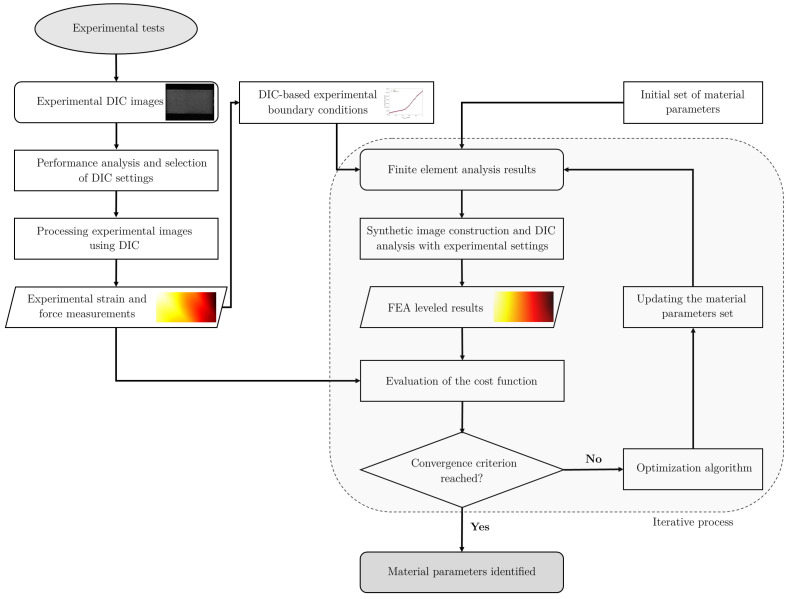
Flow diagram describing the material parameters identification process using FEMU.

The FEMU-based material parameter identification process is illustrated in Figure 1. The DIC technique is used to obtain full-field experimental measurements, which are then compared to DIC-levelled FEA results. The DIC settings are chosen by performing a parametric analysis of various sets of settings. The DIC-based experimental boundary conditions (BC) are used in the FE model. The DIC-levelled FEA results are iteratively generated by updating the material parameters set through an optimisation algorithm to minimise the discrepancies between the experimental observations and the virtual experiment results until convergence is achieved.

### 2.1. Raw Material and Specimen

**Figure 2:**
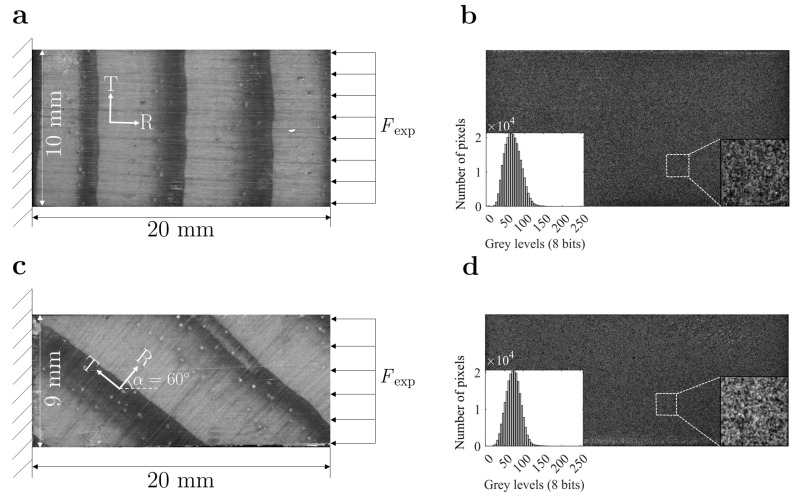
Experimental setup regarding: (**a**) Experimental boundary conditions on on-axis specimen; (**b**) Speckle pattern detail and gray level frequency histogram for one on-axis specimen; (**c**) Experimental boundary conditions on off-axis specimen; (**d**) Speckle pattern detail and gray level frequency histogram for one off-axis specimen.

Clear wood specimens were cut from a single *Pinus pinaster* tree. A radial board was selected and air-dried to a moisture content of about 12%. Wood samples were then manufactured considering the radial-tangential (RT) orthotropic orientation with nominal dimensions of 20(R) × 10(T) × 4(L) mm^3^. A total of 9 on-axis and 9 off-axis specimens were manufactured in order to compare or enhance the identifiability of the inverse material parameter identification (see Figure 2a,c for the mesostructure of one specimen of each configuration, the images with the annual growth ring structure were recorded with the same DIC optical system as presented below). An off-axis angle of 60${}^{\circ }$ was selected considering preliminary calculation based on anisotropic elasticity theory, with the criterion of balancing out both linear and shear in-plane strain components in the material coordinate system [5,46]. No specific standard test method was used, therefore the length-to-width ratio of two for the specimens was selected to prevent buckling, shear or other non-homogeneous deformation modes at the gauge section.

### 2.2. Compression Tests and Full-Field Measurements

A universal Instron 5848 MicroTester machine (Instron, Barcelona, Spain) was used to carry out the compression tests, with displacement control at a cross-head velocity of 0.5 mm/min. A 2 kN load cell was used to measure the resultant uniaxial load. To minimise friction and avoid excessive barrelling, a lubricant was applied between the specimen and the compression platens. To improve flatness of the compression surfaces, loading and unloading cycles of up to 20 N were performed before testing. Image focusing over the target pattern surface was then adjusted accordingly for stable measurements during the test.

The camera-lens optical system consisted of a Baumer Optronic FWX20 camera (Baumer Optronic GmbH, Radeberg, Germany) coupled with an AF Micro-Nikkor 200 mm f/4D ED-IF lens (Nikon, Portugal). The image field of view covered an area integrating several annual growth rings. The field of view targeted a physical region of about 20.5 × 15.5 mm^2^. The surface of the specimens was painted to carry out DIC measurements. The speckle pattern was created by means of an airbrush painting with a 0.18-mm nozzle (IWATA, model CM-B, Anesta Iwata Iberica SL, Barcelona, Spain). Figure 2b,d show the speckle pattern applied on an on-axis and off-specimen, respectively, as well as the grey level frequency histogram. The lighting system and the exposure time were set to avoid pixel saturation and image blurring during testing. Loading and image recording were synchronized during the test at an acquisition frequency of 1 Hz.

### 2.3. Digital Image Correlation: Parametric Analysis

In this work, the MatchID subset-based DIC 2D software (Ghent, Belgium [47]) was used to reconstruct the displacements and strain fields. In this technique, a mathematical correlation criterion is minimised with respect to the unknown parameters of the displacement shape function, by considering, iteratively, a sub-region centred on a pixel in the undeformed image f(x,y) and searching for the subset transformation on the deformed configuration g(x,y) [32]. A square subset can be defined by 2N+1 pixels, where *N* is a positive integer, defining the subset size (SS) or displacement spatial resolution. The size of the subsets may respect the rule of thumb of at least three contrasted pixelated speckles. On the other hand, a single speckle must contain at least three to five pixels to avoid aliasing effects. Therefore, as a first approximation, the subset size can be a multiple of three regarding the average speckle size across the image, providing that a regular pattern is created.

The zero-mean normalized sum-of-squared-differences (ZNSSD) criterion was selected mainly due to its good performance on a wide range of image contrast variations and lightning intensity shifting. DIC uses subset shape functions to match locations in the undeformed image to corresponding positions in the deformed image. The polynomial order of these shape functions determines how the subset can deform throughout the correlation process since this subset must be able to change size, shape and position during the entire deformation in order to be traced back in the deformed image. It is possible to choose between affine, irregular and quadratic shape functions, depending on the local strain gradients. Moreover, the coordinates of points in the deformed subset may be located between pixels. The intensity of these points with sub-pixel positions must be given before assessing the similarity between reference and deformed subsets using the correlation criterion, hence the need for using a sub-pixel interpolation method, such as bilinear, bicubic or bicubic spline interpolation [48].

In contrast with a finite element mesh, the correlation domains can overlap by sharing gray intensity pixels over the boundaries. The distance between adjacent centroid of the subsets is the step size (ST), which will define the mesh data points to be used in the reconstruction of the strain fields. Nevertheless, when analyzing areas with substantial heterogeneous deformation, this ST should be carefully set to achieve smooth displacement fields in particular close to holes or material transition zones.

The strain fields are then reconstructed from the displacement fields by a suitable filtering and differentiation algorithm. Typically this operation is defined locally across a strain window (SW), embedding some displacement data points N×N over which a local surface fitting approach is applied on a least-square regression sense [48]. The polynomial order of these bi-dimensional functions can be defined as bilinear or biquadratic, for instance. It is pointed out that the selected SS and ST parameters in the correlation process will propagate over the strain reconstruction by defining the final spatial resolution. This parameter can be estimated based on the following virtual strain gauge (VSG) measure (units in pixel) [47]:

(1) $$\mathrm{VSG}\;=\;\left[(\mathrm{SW}\;-\;1)\;\times \;\mathrm{ST}\right]\;+\;\mathrm{SS}.$$

Similar to SS, using a larger VSG translates to smoother results. However, the signal measured can be inaccurate in areas with high heterogeneous deformation and gradients properties. A smaller VSG, on the other hand, produces noisier results but with improved strain spatial resolution. Moreover, settings such as the SS, ST and VSG may be easily translated to physical units by means of the magnification factor of the optical system.

The selection of the DIC setting parameters is therefore critical as it influences the measurements and identification results [2]. On the one hand, using a larger SS increases the resolution, minimising the noise, but decreases the spatial resolution, which is not ideal for measuring strain gradients or heterogeneous strain fields. On the other hand, using smaller SS decreases the resolution of the measurements, however, the spatial resolution is increased.

In this work, the selection of the DIC settings was carried out with the support of the performance analysis module within MatchID [47]. This tool allows performing a large set of DIC analysis by covering a spectrum of different setting combinations at once. This analysis was systematically performed on both on-axis and off-axis specimens (Figure 2), since the off-axis angle orientation will generate a different mechanical response under the same uniaxial compressive loading. A total of 1800 analyses (900 for each specimen configuration) were performed. Table 1 reports the different parameters sets used in the parametric analysis.

**Figure 3:**
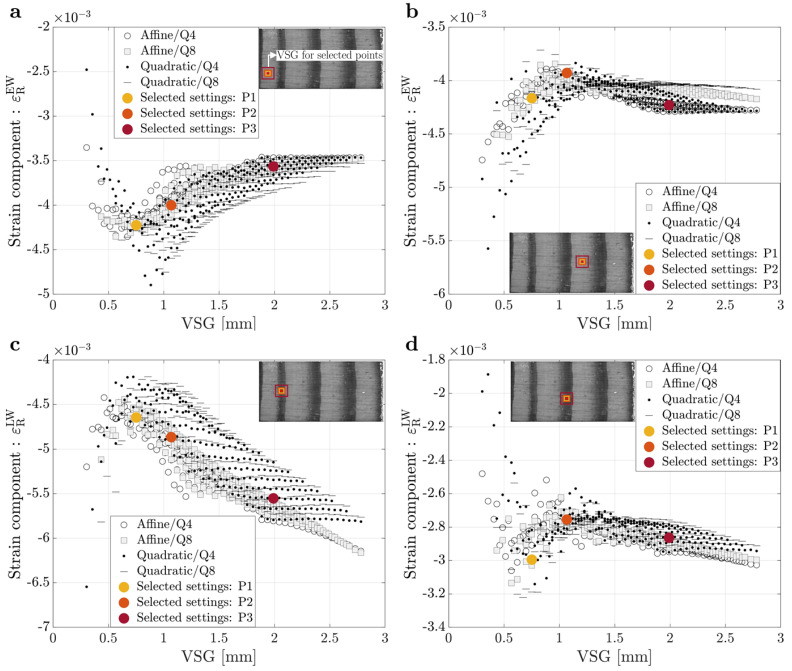
Signal versus virtual strain gauge for an on-axis specimen for different points from the ROI: (**a**) Small earlywood (EW) tissue; (**b**) Large earlywood (EW) tissue; (**c**) Small latewood (LW) tissue and (**d**) Large latewood (LW) tissue.

**Figure 4:**
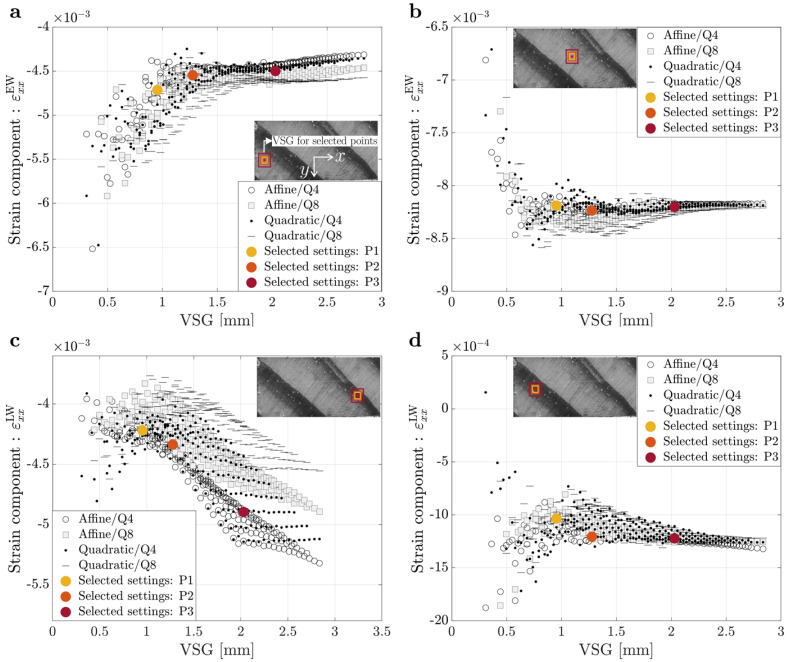
Signal versus virtual strain gauge for an off-axis specimen for different points from the ROI: (**a**) Small earlywood (EW) tissue; (**b**) Large earlywood (EW) tissue; (**c**) Small latewood (LW) tissue and (**d**) Large latewood (LW) tissue.

The sets of different DIC settings tested correspond to a VSG range between 23 and 211 pixels, or approximately 0.3 to 2.79 mm. The convergence study performed for the on-axis and off-axis specimens is summarized in Figure 3 and Figure 4, respectively, by comparing the signal measured of the strain component on the radial direction ($\varepsilon_{R}$) for the on-axis specimen and on the *x* direction ($\varepsilon_{\mathrm{xx}}$) for the off-axis specimen, with the VSG size at four different points: (a) two earlywood points and (b) two latewood points.

A larger VSG smooths the measurements, reducing the built-in noise in the experimental data, but also decreases the strain gradients that are expected in heterogeneous materials like wood, which are created by the growth ring structure [2]. Furthermore, higher VSG values appear to result in reduced magnitude of the strain signal reconstruction in the earlywood, which is to be expected given the effect of latewood tissue. When looking at the effect of VSG on strain signal reconstruction in latewood tissue, bigger values of VSG appear to lead to a higher strain signal reconstruction, in magnitude, due to the earlywood tissue influence, which is less stiff and deforms more at the same stress value. However, in some results of this parametric analysis, it is also possible to observe that the measured strain signal does not have a significant variation when the VSG increases. This is due to the VSG not being large enough to capture the transition between the earlywood and latewood tissues, which can also be confirmed by the VSG size representations found in Figure 3 and Figure 4. Furthermore, the affine and quadratic subset shape functions give comparable measurements, according to the obtained results. Likewise, bilinear and biquadratic strain interpolation, which are used to reconstruct strain fields from displacement measurements, appear to converge, suggesting that, for this analysis, the VSG size has a larger effect on the observations than these parameters. Nevertheless, these results show that when dealing with highly heterogeneous materials, it is important to carefully select the DIC setting parameters to well-balance reconstruct the strain gradients, achieving a balance between spatial resolution and accuracy.

For this work, three different sets of DIC parameters were selected for the on-axis and off-axis specimens to study the influence of spatial resolution and accuracy on the material parameters identification results, which are also represented as differently coloured dots in Figure 3 and Figure 4. The different DIC settings were selected according to different trade-offs between accuracy and spatial resolution. The first set of settings selected (P1) is characterized by good spatial resolution due to the lower value of VSG size and, consequently, adequate accuracy. The second set of settings (P2) has a lesser spatial resolution when compared to P1, but slightly improved accuracy due to the higher VSG size. Finally, the third set of setting parameters (P3) is characterized by good accuracy, owing to the high level of smoothing imposed by the larger VSG, but significantly less spatial resolution, which in turn compromises the measurement of strain gradients. Table 2 lists the 2D-DIC parameters used in this work for the on-axis and off-axis specimens, concerning the three different sets of settings chosen.

### 2.4. Finite Element Model and Synthetic Images

A FE model was implemented in ANSYS Mechanical APDL software (Pennsylvania, Canonsburg, United States of America [49]) using DIC-based experimental boundary conditions on the left and right boundaries of the region of interest (ROI), interpolated between the DIC and FEA meshes. Wood was modelled as a homogeneous orthotropic linear elastic material.

According to Hooke’s Law, if a plane stress condition is applied to an orthotropic material, the relationship between stress and strain in the global coordinate system can be expressed by [50]:

(2) $$\left[\begin{array}{c} \sigma_{x}\\ \sigma_{y}\\ \sigma_{s} \end{array}\right]\;=\;\left[\begin{array}{ccc} Q_{xx} & Q_{xy} & 0\\ Q_{xy} & Q_{yy} & 0\\ 0 & 0 & Q_{ss} \end{array}\right]\left[\begin{array}{c} \varepsilon_{x}\\ \varepsilon_{y}\\ \varepsilon_{s} \end{array}\right],$$

where $Q_{ij}$ are the stiffness matrix components in the global coordinate system, while $\sigma_{i}$ and $\varepsilon_{i}$ are the stress and strain fields, respectively, and the subscripts describe the three stress/strain components (x→xx, y→yy and s→xy). The stress/strain relationship described in Equation (2) can also be expressed regarding the modulus of elasticity, *Poisson*’s ratio and shear modulus by the following:

(3) $$\left[\begin{array}{c} \sigma_{x}\\ \sigma_{y}\\ \sigma_{s} \end{array}\right]\;=\;\left[\begin{array}{ccc} \frac{E_{x}}{1-\nu_{xy}\nu_{yx}} & \frac{-\nu_{yx}E_{x}}{1-\nu_{xy}\nu_{yx}} & 0\\ \frac{-\nu_{xy}E_{y}}{1-\nu_{xy}\nu_{yx}} & \frac{E_{y}}{1-\nu_{xy}\nu_{yx}} & 0\\ 0 & 0 & G_{xy} \end{array}\right]\left[\begin{array}{c} \varepsilon_{x}\\ \varepsilon_{y}\\ \varepsilon_{s} \end{array}\right],$$

where $E_{i}$ is the modulus of elasticity, $\nu_{ij}$ is the *Poisson*’s ratio, $G_{ij}$ is the shear modulus and the subscripts represent the different components of the global coordinate system. The *Poisson*’s ration in the different components of the global coordinate system can be further related by the following expression:

(4) $$\nu_{yx}\;=\;\nu_{xy}\frac{E_{y}}{E_{x}}.$$

As presented by Equations (3) and (4), the linear elastic orthotropic constitutive model has a total of four independent parameters to calibrate ($E_{x}$, $E_{y}$, $\nu_{xy}$ and $G_{xy}$). Moreover, the geometry of the FE models was defined considering the real rectangular shape of the specimens based on the reference experimental image. The finite element PLANE182 was selected for meshing, which is a two-dimensional four-node structural solid element. Using plane stress and pure displacement formulation, this element was defined as a plane element with two degrees of freedom at each node, which are the translations in the nodal *x* and *y* directions. The element size was set to 0.085 mm, and there were roughly 30,300 nodes and 30,000 elements on the on-axis FE models and 25,600 nodes and 25,300 elements on the off-axis FE models.

In the proposed FEMU approach, the main goal is to fit the FEA results with experimental data. However, before doing this comparison, numerous inconsistencies must be handled, including differing coordinate systems, data locations, strain formulations, spatial resolutions and data filtering. To solve these issues, it was proposed to synthetically deform the reference image of the DIC speckle pattern by means of coordinates and nodal displacements of the FE model, creating a set of deformed synthetic images for further evaluation by the DIC approach. The synthetically deformed image can then be processed using the same DIC settings as the experimental images, ensuring that both sets of data have the same filtering, spatial resolution, and strain formulation. Furthermore, using this approach guarantees that both DIC and FEA are subjected to the same calibration and triangulation processes, directly expressing both data meshes inside the same coordinate frame with coincident data point positions, avoiding further interpolation steps between the two meshes. On top of that, the experimental data are accompanied by noise and errors. However, due to this approach, which uses a real DIC reference image, numerous potential error sources associated are inadvertently included in the DIC-levelled FEA data. As a result, some pattern-related image artifacts, such as saturation, aliasing and lightning issues, may be more easily distinguished from actual model problems [44].

**Figure 5:**
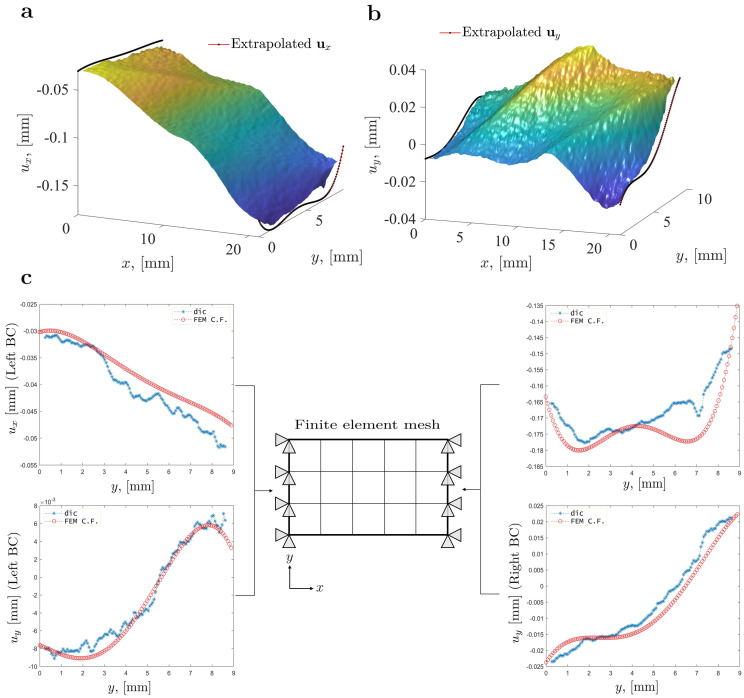
Experimental full-field displacement measurements and extrapolated boundary conditions (BC) for the FE model for an off-axis specimen on the: (**a**) *x* direction; (**b**) *y* direction; (**c**) Representation of the FE mesh and experimental boundary conditions (BC) applied to the FE model.

Following this approach, the FE model was implemented considering the whole DIC ROI. The DIC-based experimental boundary conditions were extrapolated from the experimental data points to the left and right edges of the defined ROI. Figure 5 shows the surface plot of the full-field displacement measurements along the *x* axis ($u_{x}$) and *y* axis ($u_{y}$), along with the extrapolated boundary conditions, which are applied on the FE model of one of the off-axis specimens under analysis.

This extrapolation approach is required to fully deform a region in the synthetic image that is equal to the experimental DIC ROI, thus ensuring an equal ROI on both the experimental and synthetic images, addressing the data location issue and eliminating extra interpolation processes. For the sake of simplicity, Figure 5 only shows the extrapolation results for one specimen, although this procedure was individually performed for all the specimens under analysis. The extrapolation was done considering the whole full-field displacement measurements and smoothed by a fourth-order polynomial function.

The mesh and displacement FE fields were then used to synthetically deform the experimental reference image using MatchID FE deformation module [47], as represented in the workflow in Figure 1. Afterwards, the MatchID FE validation module [47] was used to process the synthetic image, using the same DIC software and the same setting parameters used for the experimental data. This approach has increased accuracy since the discrepancies in the processing method between the two sets of data are addressed, especially when compared to the direct interpolation method [44].

### 2.5. Finite Element Model Updating Technique

The FEMU is used to find the four orthotropic linear elastic parameters of wood. An optimisation approach is used to continuously update an unknown material parameter set in order to minimise a cost function that reflects the difference between experimental measurements and FEA results. This characterisation method returns the elastic properties, which are defined by the parameter values used in the last numerical simulation of the iterative process when the minimum is reached and the optimisation process converges. Using this method, all the elastic parameters may be determined concurrently from a single experiment, given that the experimental test submits the material to a heterogeneous state of stress and strain. Displacements, stresses, loads and temperatures are all examples of data that may be used in the comparison. Because of its adaptability and simplicity of application, FEMU is a popular inverse identification approach [33,51], although the main disadvantage is the high computational time [52], which is due to the necessity for a FEA and, in this case, the generation of a synthetic image and additional processing with DIC for each objective function (OF) evaluation.

The OF used in this work describes the difference between experimental and FEA results, including the load and strain fields, and can be represented by the following expression:

(5) $$\varphi \left(\chi \right)\;=\;\left(1-W_{F}\right)IT_{S}^{2}\left(\chi \right)\;+\;W_{F}IT_{F}^{2}\left(\chi \right),\mathrm{with}\;0\;⩽\;W_{F}\;⩽\;1.$$

where χ is a vector containing the four unknown material parameters ($E_{R}$, $E_{T}$, $\nu_{\mathrm{RT}}$, and $G_{\mathrm{RT}}$) and $W_{F}$ is a weighting coefficient between the strain ($IT_{S}$) and force ($IT_{F}$) terms. The strain term is characterized as follows:

(6) $$IT_{S}\left(\chi \right)\;=\;\frac{1}{3n}\left[{\sqrt{\sum_{k\;=\;1}^{n}{\left(\frac{\varepsilon_{xx}^{\exp}\;-\;\varepsilon_{xx}^{\mathrm{num}}\left(\chi \right)}{\varepsilon_{xx,\max}^{\exp}}\right)}^{2}}}^{2}\;+\;{\sqrt{\sum_{k\;=\;1}^{n}{\left(\frac{\varepsilon_{yy}^{\exp}\;-\;\varepsilon_{yy}^{\mathrm{num}}\left(\chi \right)}{\varepsilon_{yy,\max}^{\exp}}\right)}^{2}}}^{2}\;+\;{\sqrt{\sum_{k\;=\;1}^{n}{\left(\frac{\varepsilon_{xy}^{\exp}\;-\;\varepsilon_{xy}^{\mathrm{num}}\left(\chi \right)}{\varepsilon_{xy,\max}^{\exp}}\right)}^{2}}}^{2}\right],$$

where the variable *n* is the total number of full-field measurement data points, while $\varepsilon^{\exp}$ and $\varepsilon^{\mathrm{num}}$ are the experimental and numerical strain fields, respectively, considering the different components of in-plane strain fields ($\varepsilon_{xx}$, $\varepsilon_{yy}$, and $\varepsilon_{xy}$). The variables $\varepsilon_{xx,\max}^{\exp}$, $\varepsilon_{yy,\max}^{\exp}$, $\varepsilon_{xy,\max}^{\exp}$ represent the maximum value of the experimental full-field strain measurements for each correspondent component. Moreover, the force term is defined as:

(7) $$IT_{F}\left(\chi \right)\;=\;\frac{F^{\exp}\;-\;F^{\mathrm{num}}\left(\chi \right)}{F^{\exp}}.$$

Similarly, the variables $F^{\exp}$ and $F^{\mathrm{num}}$ reflect the experimental and numerical loads for the selected stage, respectively.

To begin the iterative process of FEMU, a starting set of parameters $\chi (E_{R},E_{T},\nu_{\mathrm{RT}},G_{\mathrm{RT}})$ is given to the FEA. The numerical results are then used to generate a synthetic image, which is then processed through DIC with the same setting parameters as the experimental data, matching numerical data locations and experimental data points. The DIC-levelled FEA data are then used to evaluate the cost function and the iterative process continues, by means of an optimisation algorithm, which iteratively updates the material parameter set until a minimum of the cost function is reached.

The Nelder-Mead simplex method, which is a simple direct-search algorithm, was used in the optimisation process. In this method, a simplex is formed with as many vertices as the number of variables plus one, followed by a series of modifications aimed at minimising the OF value at its vertices [53]. The fminsearch function from MATLAB’s library (Massachusetts, Natick, United States of America [54]) was used as the optimisation technique. Moreover, the initial starting parameters were considered to be reference values [2,55] (see Table 3).

The described methodology is first validated using the DIC-levelled FEA results as our reference in the FEMU technique (Section 3.1). With this approach, an exact solution for the constitutive parameters is known, and expected to be identified, being the reference parameters given to the FE model (the case of virtual experiments). Moreover, the $W_{F}$ used in this method validation approach is $10^{-10}$. The $W_{F}$ value used on the experimental identification is $10^{-1}$, which privileges the strain term minimisation, while also being capable of minimising the difference between the numerical and experimental loads, as is shown on the convergence study for the identified parameters (Section 3.3). The difference between the $W_{F}$ used in both cases is due to the $IT_{S}$ value, which is lower in the method validation, since we are using the DIC-levelled FEA results as our reference, and therefore the FE model should be able to precisely reproduce the same results in the parameters identification procedure. The $IT_{S}$ on the experimental identification is higher since there are differences between the experimental observations and the numerical results, coming from the constitutive model used in the FEA, and in this case also owing to the homogeneous modelling of wood used in this work. The $W_{F}$ determines a balance between the strain and forces terms, and the goal is to minimise the difference between the strain fields, while also being able to minimise the differences between the experimental and numerical loads. If the $IT_{S}$ value is low (which is the case for the virtual experiments validation, see Section 3.1), it means that the $W_{F}$ value has also to be low. A lower $W_{F}$ value gives more weight to the strain fields differences minimisation and allows the $IT_{S}$ to converge to a lower value than the $IT_{F}$, while also allowing the optimisation method to more easily reach a global minimum instead of a local minimum. However, the $W_{F}$ should be carefully selected, because if an overly low value is used for this weight, the force term may not be minimised. As a result, the difference between the experimental and numerical loads should be verified alongside the identification results.

Furthermore, as illustrated in Figure 2, on the off-axis specimens, the global coordinate systems and fibre coordinates systems are rotated by the off-axis angle (α). This angle was measured for each of the off-axis specimens under analysis and taken into account in the rotation matrix between the two coordinate systems. Therefore, the constitutive parameters of the off-axis specimens were identified on the fibre orientation coordinate system, thus comparable to the reference parameters and the values identified for the on-axis specimens.

Moreover, when the load is increased, the measured displacements rise as well, which improves the signal-to-noise ratio and hence the identification. However, the load cannot be raised above a certain point without deviating from linear elastic behaviour. Therefore, the stage selection for the identification process was performed based on this premise, by selecting a later stage, in order to achieve a good signal-to-noise ratio, while also making sure that the material is still undergoing linear elastic deformation.

## 3. Results

### 3.1. Method Validation

The validation of the described methodology was carried out for the two specimen configurations (on-axis and off-axis specimens) by running a FEA with the experimental boundary conditions and the reference parameters for this wood species. Then, using nodal displacements and mesh information from the FEA, a synthetic image was generated, which was processed by DIC using the P1 settings described in Table 2. These results were then used as the reference in the identification procedure. To evaluate the convergence to the known solution, the starting parameters given to the FE model at the start of the iterative process deviate from the reference parameters (starting parameters used: $E_{R}$ = 1298 MPa; $E_{T}$ = 548 MPa; $\nu_{\mathrm{RT}}$ = 1; $G_{\mathrm{RT}}$ = 211 MPa).

Table 4 summarizes the final OF, strain and force terms values, as well as the comparison between the reference and the final calibrated numerical force for the two specimen configurations.

**Figure 6:**
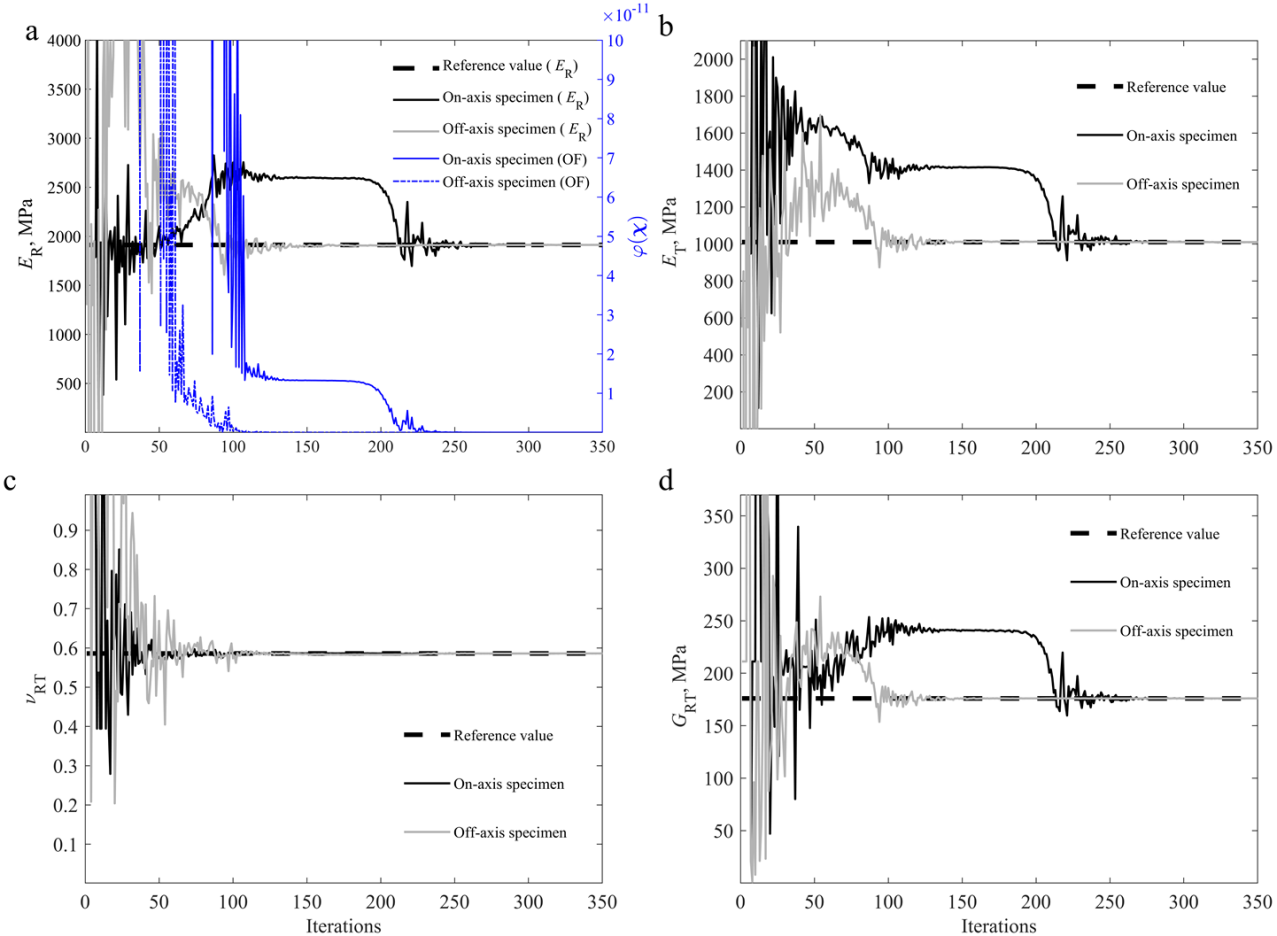
Convergence study for an on-axis specimen and off-axis specimen using a DIC-levelled FEA reference with reference parameters [2,55], regarding: (**a**) $E_{R}$ and $\varphi \left(\chi \right)$; (**b**) $E_{T}$; (**c**) $\nu_{\mathrm{RT}}$; (**d**) $G_{\mathrm{RT}}$.

The differences between the reference and numerical strain fields and loads are minimised, with the linear elastic orthotropic constitutive parameters being the only variables to be determined in the optimisation procedure. Figure 6 shows the convergence study for all four material parameters identified ($E_{R}$, $E_{T}$, $\nu_{\mathrm{RT}}$, and $G_{\mathrm{RT}}$) during the identification process for both specimen configurations.

Table 5 shows the identification results for the four material parameters. The results obtained validate the methodology applied since the inverse identification procedure was able to converge to the known solution, with errors of 0% for both specimen configurations.

### 3.2. Influence of the DIC Settings on the Identified Parameters

**Figure 7:**
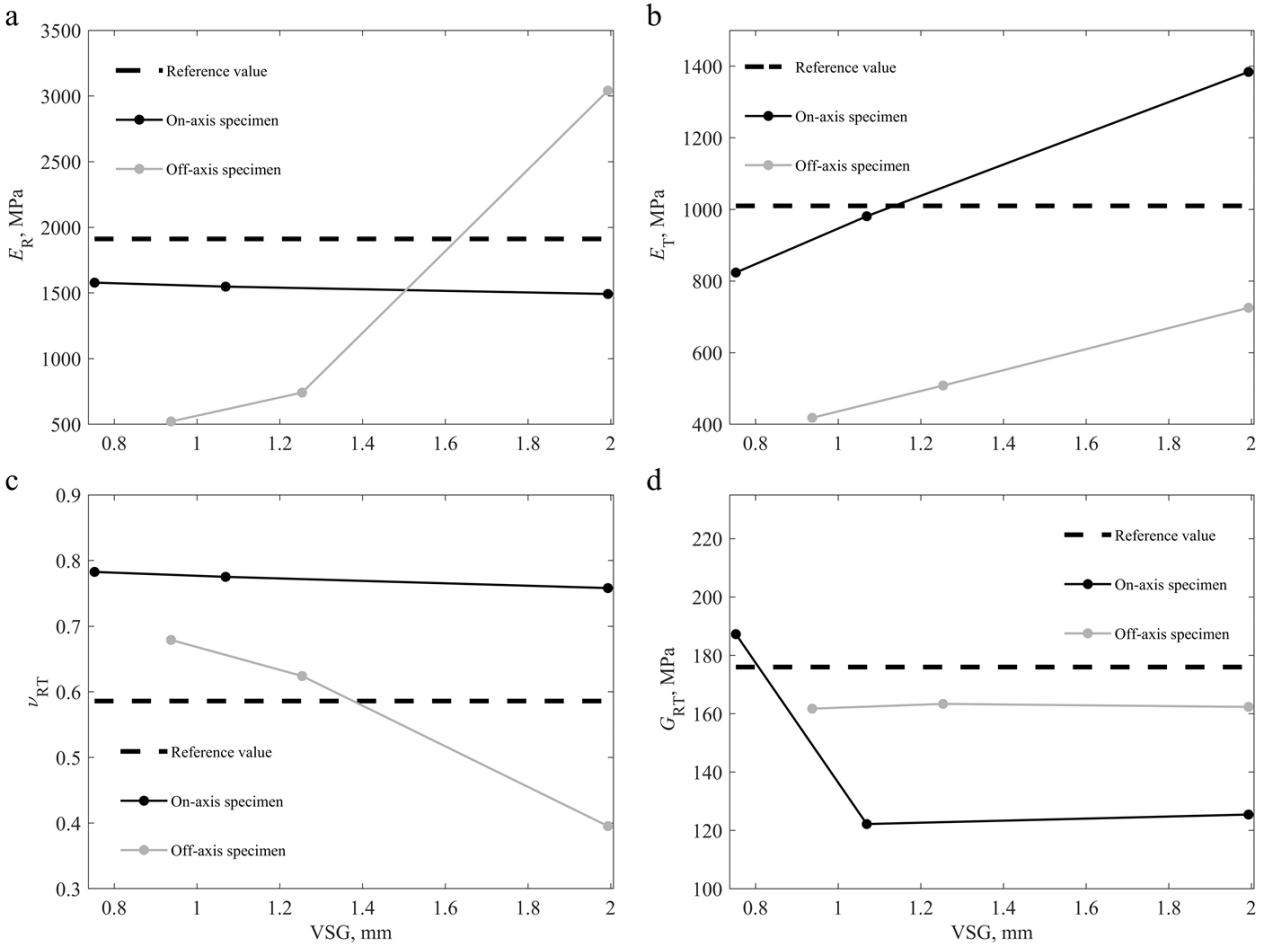
Results for the identification process for an on-axis and off-axis specimens using three different DIC settings (Table 2), and compared to the reference values [2,55], regarding the identified parameters: (**a**) $E_{R}$; (**b**) $E_{T}$; (**c**) $\nu_{\mathrm{RT}}$; (**d**) $G_{\mathrm{RT}}$.

The methodology was further extended to the experimental data, investigating how DIC settings affect the identification results for two specimens with different specimen configurations. The identification was performed on one on-axis and one off-axis specimens using the DIC setting parameters summarised in Table 2. Figure 7 describes the obtained results.

On the on-axis specimen, the variation of the $E_{R}$ and $\nu_{\mathrm{RT}}$ appears to be small as the VSG size increases. However, on the off-axis specimen, the variation for these parameters appears to be larger as the VSG size changes. On the other hand, the variation of $G_{\mathrm{RT}}$ is lower on the off-axis specimen and higher on the on-axis specimen, whereas the $E_{T}$ varies almost linearly on both specimen configurations. Theoretically, the modulus of elasticity in the radial direction ($E_{R}$) is the parameter with the most identifiability on the on-axis specimen, given the test configuration used. Similarly, the shear modulus of the RT plane ($G_{\mathrm{RT}}$) is, theoretically, the most identifiable parameter for the off-axis specimen. These results suggest that DIC settings have less impact on parameters with high identifiability. Nonetheless, there are still differences that have a particular impact on the identification of heterogeneous material properties. The identification results for the various VSG sizes tested are shown in Table 6 for both specimen configurations.

The DIC settings influence the amount of smoothing introduced into the results, averaging the measurements in a given VSG. For the tested wooden specimens, the volume fraction of earlywood tissue was greater than latewood. It is noticed that the elastic properties of latewood are greater than that of earlywood. When the measurements are averaged, the results are expected to be influenced primarily by the material with the highest volume fraction. As a result, as the VSG size increases, the identified value for the $E_{R}$ for the on-axis specimen decreased, averaging out the results and resulting in the loss of strain gradients.

Wood is a natural material with a high degree of natural variability. Therefore, the experimental and identification procedures were carried out on a total of 18 specimens (9 on-axis specimens and 9 off-axis specimens), in order to conduct a statistical analysis of the identification results. For the remainder of this work, the P1 DIC settings from Table 2 were used, since these settings allow for the measurement of strain gradients, which is especially important when identifying constitutive parameters of heterogeneous materials.

### 3.3. Convergence Study for Identified Parameters

The differences between the full-field experimental strain measurements and numerical strain, as well as the difference between the experimental and numerical loads are minimised, where the optimisation variables are the material constitutive parameters ($E_{R}$, $E_{T}$, $\nu_{\mathrm{RT}}$, $G_{\mathrm{RT}}$). Table 7 outlines the final OF, strain and force term values, and also the comparison between experimental force measurement and final calibrated numerical force for the 18 specimens analysed.

**Figure 8:**
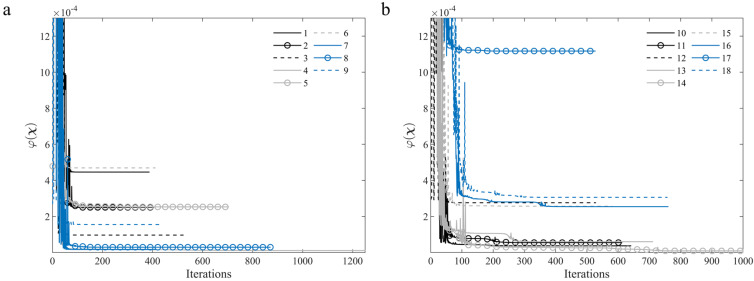
Convergence of the OF value during the identification process for: (**a**) On-axis (1–9) specimens and (**b**) Off-axis (10–18) specimens.

**Figure 9:**
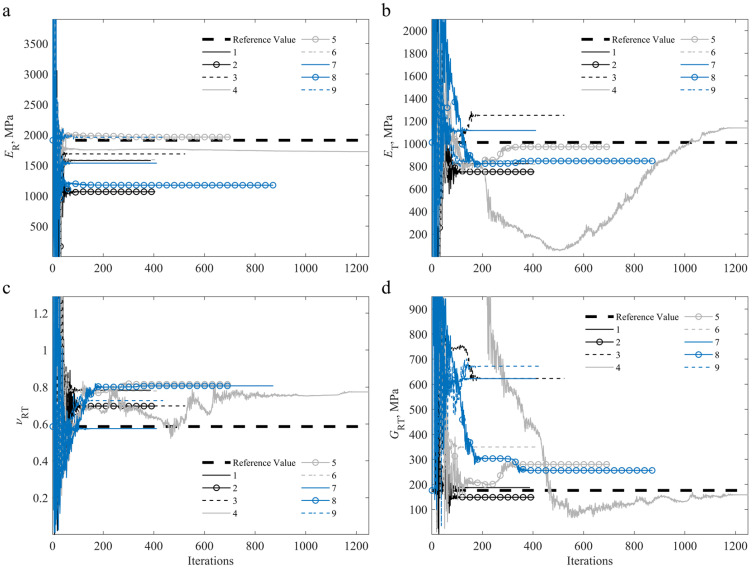
Convergence of the identified parameters for the on-axis (1–9) specimens during the iterative procedure, compared to the reference values [2,55]: (**a**) $E_{R}$; (**b**) $E_{T}$; (**c**) $\nu_{\mathrm{RT}}$; (**d**) $G_{\mathrm{RT}}$.

**Figure 10:**
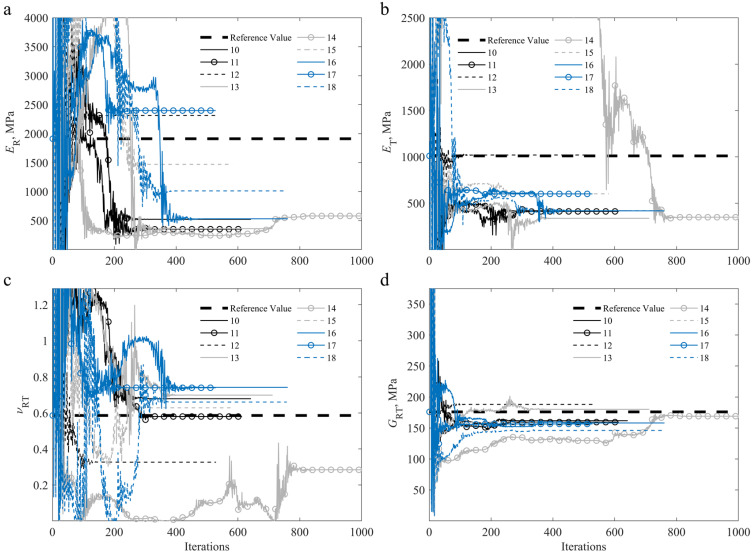
Convergence of the identified parameters for the off-axis (10–18) specimens during the iterative procedure, compared to the reference values [2,55]: (**a**) $E_{R}$; (**b**) $E_{T}$; (**c**) $\nu_{\mathrm{RT}}$; (**d**) $G_{\mathrm{RT}}$.

From Figure 8, Figure 9 and Figure 10, it can be seen that the identification of the parameters of specimens 4 and 14 proved to be more time-consuming in terms of computational time, requiring close to 1200 (specimen 4) and 1000 (specimen 14) iterations to reach the minimum and for the process to stagnate. It is also worth noting that the $E_{R}$ was the overall most stable parameter throughout the identification process, whereas the $G_{\mathrm{RT}}$ was the most stable parameter for the off-axis specimens.

### 3.4. Experimental and Numerical Full-Field Strain Maps

**Figure 11:**
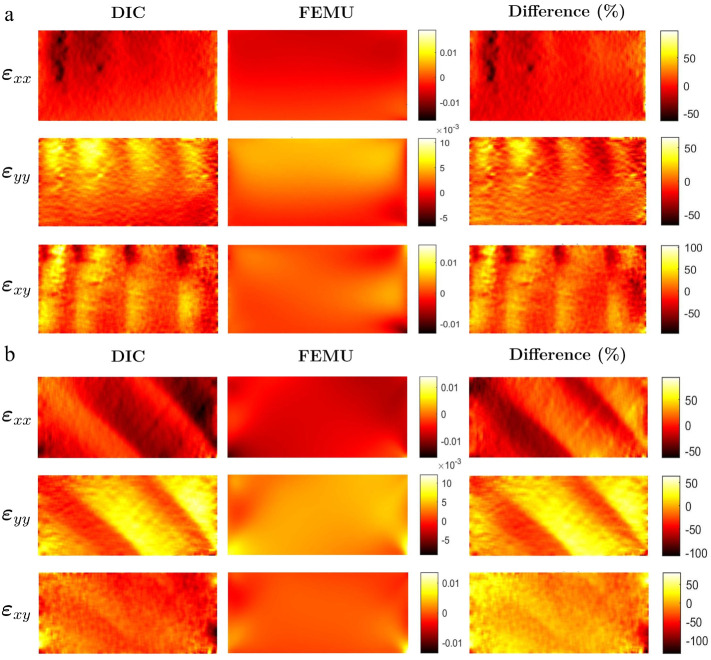
Experimental calibrated numerical and difference full-field strain maps for: (**a**) Specimen 1 and (**b**) Specimen 17.

The numerical strain maps are reconstructed over the same filtering as the experimental DIC full-field measurements. This allows for a fair comparison of strain maps and inspecting the differences that come out from constitutive model issues, as well as the differences resulting from the homogeneous modelling of wood used in this work. Figure 11 shows the experimental DIC strain fields in comparison to the final calibrated numerical strain fields for both on-axis and off-axis specimens. Moreover, the residual differences between numerical and experimental strain fields, normalized by the maximum value of strain of each correspondent component, are also plotted. The residual maps show a systematic pattern related to the fact that the finite element model was built under the assumption of a homogeneous material, while experimentally, at the scale of observation, the annual rings morphology generate a heterogeneous strain map due to local stiffness difference between the wood meso layers.

### 3.5. Identified Orthotropic Linear Elastic Parameters

The orthotropic linear elastic constitutive parameters of *Pinus pinaster* for the RT plane are identified. The constitutive parameters were determined for each on-axis and off-specimen and are listed in Table 8 and Table 9, respectively, and include the average value, standard deviation (Sdt), and coefficient of variation (CoV).

The results show some dispersion, which is to be expected given wood intrinsic natural variability. The average values of the modulus of elasticity identified for the on-axis specimens on the radial and tangential directions, as well as the *Poisson*’s ratio on the RT plane, agree with the reference values for this wood species, while also showing a CoV inferior to 20%.

The off-axis specimen identification results, on the other hand, showed an exceptionally consistency on the shear modulus on the RT plane, with a CoV less than 10% and an average value that agrees with the reference value reported in the literature. The *Poisson*’s ratio on the RT plane also agrees with the reference value, although with a higher dispersion (CoV of 28.86%).

## 4. Discussion

The proposed FEMU methodology, which is based on a synthetic image approach and uniaxial compression tests, while using on-axis specimens proved to be effective in the identification of three out of four RT orthotropic linear elastic constitutive parameters of *Pinus pinaster*, which were the modulus of elasticity in the radial and tangential directions and the *Poisson*’s ratio on the RT plane. The mean value for these parameters can be compared to the typical values reported in the literature, with a CoV ranging from 10.2% to 19.4%. The shear modulus identified using the on-axis configuration has a higher dispersion with a CoV of 58.5%. Furthermore, for the identified mean values, the anisotropy ratio on the RT plane, which is determined by the ratio between $E_{R}$ and $E_{T}$, is 1.67, which is comparable to the values reported in the literature [6].

Moreover, the proposed approach was successful in identifying the shear modulus of the RT plane on off-axis specimens. The average identified value of this parameter agrees with the reference value and has a low CoV of 7.8%. While the remaining parameters show a higher dispersion with a CoV in between 28.9% and 77.0%. These results show that due to the lack of sufficiently heterogeneous strain fields, there is a dependency on the test configuration and the identifiability of some material parameters.

Some of the dispersion found in the results is most likely due to variations in material properties between specimens. It should be noted that the specimens used in this work were manufactured from a different tree and position within the stem than specimens tested to identify reference values, therefore some variability in elastic constants is to be expected [6].

## 5. Conclusions

An inverse identification strategy based on FEMU was proposed in this work to identify orthotropic elastic properties of wood from a single test configuration. The advantage of the proposed approach, with regard to common optimisation, was that the algorithm had been designed to evaluate a cost function based on numerical and experimental strain fields that were reconstructed from the same DIC filtering computation workflow. The approach was applied to the material parameters identification of *Pinus pinaster* wood on the radial-tangential plane. The methodology to simultaneously identify the linear elastic orthotropic constitutive parameters was based on uniaxial compression tests with on-axis and off-axis specimens. A synthetic image approach based on DIC was coupled to FEMU to identify the parameters of the constitutive model. The following main conclusions can be drawn from this study:

- The proposed methodology using a DIC-levelled FEA reference (virtual experiment) in the identification procedure was successfully validated. The iterative process of FEMU was also coupled to synthetic image generation, taking into consideration the FEA nodal displacements and mesh information.
- For each specimen configuration, a convergence study of the DIC settings was systematically carried out. The effect of the selected DIC parameters on the identification results was evaluated. When the material parameter was well identified, the DIC settings had no significant influence on the convergence. However, when the elastic parameters were less sensitive to the identification, this influence was higher.
- The average values identified on the on-axis specimens for the modulus of elasticity on the radial and tangential directions, as well as the *Poisson*’s ratio of the RT plane, show an agreement to the reference value and a lower variation when compared to the values identified for the shear modulus.
- On the off-axis specimens, the shear modulus of the RT plane agrees with the reference value, while also showing a low variation, with a CoV of 7.82%. Given the natural variability of natural materials such as wood, the scatter in the identification results is to be expected.
- The results show that three out of four RT linear elastic orthotropic parameters of *Pinus pinaster* were identified based on an on-axis specimen configuration ($E_{R}$, $E_{T}$ and $\nu_{\mathrm{RT}}$), and one of the four parameters was correctly identified when using off-axis specimen configuration ($G_{\mathrm{RT}}$).
- Other heterogeneous test configurations should be investigated in future work to increase the identifiability of all constitutive parameters using a single test. Furthermore, this methodology may be used to identify the heterogeneous orthotropic constitutive properties of wood.

## Figures and Tables

**Table 1:** Different combinations of DIC parameters set used on the parametric analysis.

Correlation Settings	On-Axis Specimen	Off-Axis Specimen
Subset size	{9–81 px}, 4 px increment	{9–81 px}, 4 px increment
Step size	{5 px}, fixed	{5 px}, fixed
Shape function	{Affine, quadratic}	{Affine, quadratic}
Strain window size	{3–27}, 2 point increment	{3–27}, 2 point increment
Strain interpolation	{Bilinear Q4, Biquadratic Q8}	{Bilinear Q4, Biquadratic Q8}
Strain convention	{Green–Lagrange}	{Green–Lagrange}

**Table 2:** 2D-DIC settings used for the experimental DIC measurements using MatchID DIC Software [47].

2D-DIC Setting Parameters	On-Axis Specimens		Off-Axis Specimens
Camera	Baumer Optronic FWX20		
Lens	AF Micro-Nikkor 200 mm f/4D ED-IF		
Field of view	21.5 × 16.5 mm${}^{2}$		
Image conversion factor	0.0132 mm/px		
Working distance	721 mm		
Image aquisition frequency	1 Hz		
Speckle pattern technique	Airbrush painting		
Average speckle size	2.69 px/0.036 mm	|	4 px/0.053 mm
Image resolution	1624 × 1236 px${}^{2}$		
Correlation criterion	ZNSSD		
Interpolant	Bicubic spline		
Subset shape function	Quadratic		
Subset size	17 px (P1), 21 px (P2), 41 px (P3)	|	21 px (P1), 25 px (P2), 41 px (P3)
Step size	5 px		
Image pre-filtering	Gaussian, 5 px kernel		
Strain window size	9 (P1), 13 (P2), 23 (P3)	|	11 (P1), 15 (P2), 23 (P3)
Strain interpolation	Bilinear Q4		
Strain convention	Green–Lagrange		

**Table 3:** Reference constitutive parameters for *Pinus pinaster* [2,55].

	$E_{R}$ (MPa)	$E_{T}$ (MPa)	$\nu_{\mathrm{RT}}$	$G_{\mathrm{RT}}$ (MPa)
Reference parameters	1912	1010	0.586	176

**Table 4:** Summary of FEMU parameters on the final iteration, for an on-axis and off-axis specimens using a DIC-levelled FEA reference.

Specimen	$\varphi \left(\chi \right)$	${\mathrm{IT}}_{S}\left(\chi \right)$	${\mathrm{IT}}_{F}\left(\chi \right)$	$F^{\mathrm{ref}}$ [N]	$F^{\mathrm{num}}\left(\chi \right)$ [N]
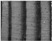	$2.59\times 10^{-22}$	$1.61\times 10^{-11}$	$1.68\times 10^{-9}$	326.4	326.4
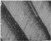	$8.45\times 10^{-25}$	$9.13\times 10^{-13}$	$1.05\times 10^{-8}$	128.4	128.4

**Table 5:** Identification results for an on-axis specimen and an off-axis specimen, using a DIC-levelled FEA reference with reference parameters [2,55].

		$E_{R}$ (MPa)	$E_{T}$ (MPa)	$\nu_{\mathrm{RT}}$	$G_{\mathrm{RT}}$ (MPa)
Reference Parameters		1912	1010	0.586	176
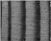	Id. value	1912	1010	0.586	176
	Error (%)	0.000	0.000	0.000	0.000
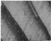	Id. value	1911.9	1010	0.586	176
	Error (%)	0.005	0.000	0.000	0.000

**Table 6:** Results from the experimental identification for an on-axis specimen and off-axis specimens, using different DIC settings and compared to the reference parameters [2,55].

	VSG (mm)	$E_{R}$ (MPa)	$E_{T}$ (MPa)	$\nu_{\mathrm{RT}}$	$G_{\mathrm{RT}}$ (MPa)
Reference Parameters		1912	1010	0.586	176
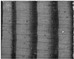	0.7524	1578.8	823.7	0.783	187.3
	1.0692	1548.5	981	0.775	122.2
	1.9932	1492.5	1384.1	0.758	125.5
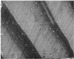	0.9372	521.5	418.2	0.679	161.7
	1.254	741.7	508.2	0.624	163.4
	1.9932	3041.5	725.4	0.395	162.3

**Table 7:** Summary of the OF terms at the end of the identification procedure using FEMU.

Orientation	Specimen	$\varphi \left(\chi \right)$	${\mathrm{IT}}_{S}\left(\chi \right)$	${\mathrm{IT}}_{F}\left(\chi \right)$	$F^{\exp}$ (N)	$F^{\mathrm{num}}\left(\chi \right)$ (N)
	1	$4.462\times 10^{-4}$	$2.227\times 10^{-2}$	$3.737\times 10^{-5}$	273.4	273.4
	2	$2.505\times 10^{-4}$	$1.668\times 10^{-2}$	$1.402\times 10^{-5}$	314.0	314.0
	3	$9.682\times 10^{-5}$	$1.037\times 10^{-2}$	$2.198\times 10^{-4}$	298.8	298.8
On-axis	4	$1.072\times 10^{-5}$	$3.451\times 10^{-3}$	$1.514\times 10^{-4}$	148.9	148.9
specimens	5	$2.531\times 10^{-4}$	$1.676\times 10^{-2}$	$1.332\times 10^{-3}$	327.6	327.2
	6	$4.694\times 10^{-4}$	$2.284\times 10^{-2}$	$1.768\times 10^{-4}$	295.4	295.4
	7	$1.710\times 10^{-5}$	$4.359\times 10^{-3}$	$6.874\times 10^{-5}$	137.2	137.2
	8	$2.957\times 10^{-5}$	$5.732\times 10^{-3}$	$4.127\times 10^{-5}$	120.6	120.6
	9	$1.554\times 10^{-4}$	$1.314\times 10^{-2}$	$1.245\times 10^{-5}$	404.3	404.3
	10	$3.718\times 10^{-5}$	$6.427\times 10^{-3}$	$9.683\times 10^{-6}$	106.0	106.0
	11	$5.516\times 10^{-5}$	$7.829\times 10^{-3}$	$1.406\times 10^{-5}$	100.1	100.1
	12	$2.762\times 10^{-4}$	$1.752\times 10^{-2}$	$1.163\times 10^{-5}$	101.6	101.6
Off-axis	13	$6.004\times 10^{-5}$	$8.168\times 10^{-3}$	$3.317\times 10^{-5}$	73.7	73.7
specimens	14	$9.178\times 10^{-6}$	$3.193\times 10^{-3}$	$1.890\times 10^{-5}$	73.7	73.7
	15	$2.571\times 10^{-4}$	$1.690\times 10^{-2}$	$9.720\times 10^{-5}$	97.7	97.7
	16	$2.544\times 10^{-4}$	$1.681\times 10^{-2}$	$3.837\times 10^{-5}$	99.1	99.1
	17	$1.117\times 10^{-3}$	$3.523\times 10^{-2}$	$1.132\times 10^{-4}$	104.0	104.0
	18	$3.063\times 10^{-4}$	$1.845\times 10^{-2}$	$9.825\times 10^{-5}$	102.5	102.5

**Table 8:** Identified orthotropic linear elastic parameters for the on-axis specimens and comparison to the reference values [2,55].

	$E_{R}$ (MPa)	$E_{T}$ (MPa)	$\nu_{\mathrm{RT}}$	$G_{\mathrm{RT}}$ (MPa)
Reference Parameters	1912	1010	0.586	176
Specimen 1	1578.8	823.7	0.783	187.3
Specimen 2	1065.1	749.9	0.697	148.6
Specimen 3	1687.0	1250.3	0.698	623.4
Specimen 4	1725.4	1138.2	0.774	158.3
Specimen 5	1964.2	971.0	0.815	280.0
Specimen 6	1591.4	817.6	0.779	349.4
Specimen 7	1535.8	1116.1	0.575	622.8
Specimen 8	1173.6	846.1	0.807	255.9
Specimen 9	1961.4	845.7	0.727	671.7
Average	1587.0	951.0	0.739	366.4
Sdt	307.7	176.4	0.076	214.4
CoV	19.39%	18.55%	10.22%	58.53%

**Table 9:** Identified orthotropic linear elastic parameters for the off-axis specimens and comparison to the reference values [2,55].

	$E_{R}$ (MPa)	$E_{T}$ (MPa)	$\nu_{\mathrm{RT}}$	$G_{\mathrm{RT}}$ (MPa)
Reference Parameters	1912	1010	0.586	176
Specimen 10	521.5	418.2	0.679	161.7
Specimen 11	352.0	411.1	0.580	159.5
Specimen 12	2313.1	1019.6	0.327	188.1
Specimen 13	359.9	337.1	0.699	180.1
Specimen 14	579.6	348.5	0.284	169.0
Specimen 15	1469.9	601.0	0.629	160.0
Specimen 16	530.7	417.3	0.742	158.1
Specimen 17	2398.1	600.2	0.740	156.5
Specimen 18	1011.8	418.6	0.661	146.0
Average	1059.6	507.9	0.593	164.3
Sdt	815.7	214.2	0.171	12.8
CoV	76.99%	42.18%	28.86%	7.82%

## Data Availability

Not applicable.
